# Vocal Cord Dysfunction in Nonlaryngeal Head and Neck Cancer After Chemoradiation Therapy: Predictive Modeling Using CT Radiomics and Machine Learning

**DOI:** 10.1155/bmri/1246604

**Published:** 2025-11-29

**Authors:** Sakineh Bagherzadeh, Pedram Fadavi, Hamid Abdollahi, Amir Mohamad Arefpour, Mahdi Asgari, Foad Goli Ahmadabad, Mojtaba Safari, Manijeh Beigi

**Affiliations:** ^1^ Department of Medical Physics, School of Medicine, Semnan University of Medical Sciences, Semnan, Iran, semums.ac.ir; ^2^ Department of Radiation Oncology, School of Medicine, Shohadaye Haftom-e-Tir Hospital, Iran University of Medical Sciences, Tehran, Iran, iums.ac.ir; ^3^ Department of Radiology, University of British Columbia, Vancouver, British Columbia, Canada, ubc.ca; ^4^ Department of Integrative Oncology, BC Cancer Research Center, Vancouver, British Columbia, Canada; ^5^ Department of Radiation Oncology, School of Medicine, Firouzgar Hospital, Iran University of Medical Sciences, Tehran, Iran, iums.ac.ir; ^6^ Department of Medical Physics, School of Medicine, Kermanshah University of Medical Science, Kermanshah, Iran, kums.ac.ir; ^7^ Department of Physics, Engineering Physics and Optics, and Cancer Research Centre, Laval University, Quebec City, Canada, ulaval.ca

**Keywords:** chemoradiation therapy, machine learning, radiomics, vocal cord dysfunction

## Abstract

**Introduction:**

This study aims to investigate computed tomography (CT) radiomic features and dosimetric–clinical biomarkers to predict vocal cord dysfunction (VCD) in nonlaryngeal head and neck cancer (HNC) patients treated with chemoradiation therapy (CRT), using machine learning (ML) models.

**Methods:**

Sixty‐five HNC patients who underwent CRT were recruited to assess radiation‐induced VCD 6 months posttreatment. For each patient, CT radiomic features of the laryngeal region, clinical, and dose–volume histogram (DVH) metrics were collected to develop ML models. Nine classifiers were trained using selected features obtained from three feature selection algorithms: least absolute shrinkage and selection operator (LASSO), extra trees, and elastic net. The models were built using imaging features alone (radiomics model) and in combination with clinical and dosimetric features (combined model). Model performance was evaluated using the area under the receiver operating characteristic curve (AUC‐ROC).

**Results:**

Of the 65 patients, 31 developed VCD. Among radiomics models, the AdaBoost and random forest (RF) classifiers performed best, with AUCs of 0.74 and 0.84, respectively. For the combined models, nine classifiers achieved an AUC greater than 0.95 using LASSO and elastic net algorithms. In contrast, only one classifier surpassed an AUC of 0.95 when using the extra trees algorithm.

**Conclusion:**

Our findings demonstrate that pretreatment CT radiomic features are predictive biomarkers for radiation‐induced toxicities, including VCD. Furthermore, combining radiomic features with clinical and dosimetric data can improve the predictive modeling of radiotherapy outcomes.

## 1. Introduction

Head and neck cancers (HNCs) rank as the seventh most common malignancy worldwide, with approximately 890,000 new cases and 450,000 deaths reported annually across the globe [1]. Chemoradiation therapy (CRT) has emerged as a promising treatment for HNCs. However, treatment toxicity is one of the most important factors to consider when evaluating the benefit/risk ratio while deciding on a treatment strategy for HNC radiotherapy [2–4]. In many studies, radiation therapy (RT) outcomes and treatment‐related toxicities in HNC have been quantitatively defined using tumor control probability (TCP) and normal tissue complication probability (NTCP). In addition to these radiobiological models, machine learning (ML) techniques have been applied to improve the prediction of radiation‐induced side effects in HNC patients [5–7]. ML methods enable the integration of large and diverse datasets, including clinical and dosimetric information, which can be combined with quantitative data extracted from medical images, such as radiomics. Radiomics is an advanced image‐processing technology that analyzes medical images and converts them into biomarkers to support clinical decision‐making, treatment planning, and toxicity assessment. Computed tomography imaging is routinely used in the management of HNC for diagnosis, radiation treatment planning, and posttreatment surveillance. As a result, radiomics provides significant clinical value by enabling additional diagnostic insights without requiring further imaging procedures [5, 7–9]. For instance, one study used radiomics features derived from cochlear CT to predict chemoradiotherapy‐induced sensorineural hearing loss in HNC patients [10]. Another study developed an acute xerostomia system based primarily on changes in radiomics from CT images for patients with nasopharyngeal cancer (NPC) during radiotherapy [11]. Beyond these toxicities, RT for HNC frequently leads to subtle changes in the laryngeal mucosa, laryngeal edema, and vocal cord dysfunction (VCD), which are among the predominant side effects affecting the larynx. The severity of these complications is influenced by both the total radiation dose and the duration of exposure [12–17]. This study is aimed at developing predictive models for radiotherapy‐induced VCD in patients with nonlaryngeal HNCs by incorporating CT radiomic features, clinical variables, and dosimetric parameters assessed 6 months postradiotherapy. To the best of our knowledge, the integration of CT‐derived biomarkers with clinical and dosimetric features in prognostic models for radiotherapy‐induced VCD has not been previously explored.

## 2. Materials and Methods

### 2.1. Patients

This prospective study was approved by the institutional review board, and written informed consent was obtained from all participants. The study included 65 patients with nonlaryngeal HNCs and 80 healthy control volunteers. The control group was age‐ and sex‐matched to the patient cohort (mean age 50.80 ± 12.48, with 56% male and 44% female). None of the control participants reported smoking history, chronic respiratory disease, or professional voice use (e.g., singers, teachers, and broadcasters). All controls reported normal voice function with no prior laryngeal pathology. Recruitment details of the patient group are summarized in Table 1. Patients underwent the RT with total doses ranging from 45 to 70 Gy, administered in 25–35 fractions. Approximately 28% of the patients (23 males and 14 females) received concurrent chemotherapy, consisting of weekly doses of cisplatin (40 mg/m^2^; Bristol Myers Squibb). All patients underwent CT imaging in the treatment position before radiotherapy, with scans acquired at 100 kV, 220 mAs, and a slice thickness of 3 mm.

**Table 1:** Patients′ characteristics and demographics data.

**Nonlaryngeal patients**	**Frequency (%)**
Number of patients	65 (100%)
Mean age (range)	50.31 ± 16.24 (15–82)
Males	40 (61.53%)
Females	25 (38.46%)
Site of tumors	
Nasopharyngeal	24 (36.92%)
Oral cavity	17 (26.15%)
Non‐Hodgkin lymphoma	14 (21.53%)
Parotid	10 (15.38%)
Smoking status	
Males	27 (67.5%)
Females	4 (16%)
Types of treatment	
Radiation therapy	28 (43.07%)
Chemoradiation therapy (CRT)	37 (56.92%)

### 2.2. Voice Assessment

Patients participating in the study were instructed to prolong the vowel /a/ in their habitual tone for 5 s while seated in a soundproof room, to obtain voice signals for analysis. The voice signals were recorded using a certified recorder (Zoom, H5, Japan) at a sampling frequency of 44.1 kHz. From each recording, a 3‐s segment of sustained voice was selected for analysis, and acoustic features were extracted using PRAAT software (Version 6.0.25). Voice recordings were obtained at three specific time points for each patient: before treatment (baseline), at the end of treatment, and 6 months posttreatment [18]. Acoustic features were extracted from both the patient and control groups, and key parameters—including fundamental frequency (F0), jitter, shimmer, and harmonics‐to‐noise ratio (HNR)—were compared. Patients were included in the study only if their baseline values for these measures fell within one standard deviation of the control group′s mean [19].

### 2.3. Radiomic Workflow

The larynx region was manually contoured on all CT slices by an experienced radiation oncologist, and the contours were subsequently reviewed by a second radiation oncologist. A total of 107 radiomic biomarkers were then extracted from the manually contoured larynx regions using the PyRadiomics package, following the guidelines of the Image Biomarker Standardization Initiative. These radiomic features included 19 first‐order statistics features, 16 three‐dimensional (3D) shape–based features, 24 gray‐level co‐occurrence matrix (GLCM) features, 16 gray‐level run length matrix (GLRLM) features, 16 gray‐level size zone matrix (GLSZM) features, five neighbor gray‐tone difference matrix (NGTDM) features, and 14 gray‐level dependence matrix (GLDM) features.

### 2.4. Feature Selection and Classification

Three feature selection methods were used to identify a subset of radiomic, clinical, and dosimetric characteristics that offered better predictive power while minimizing computational burden and processing time. These methods included the extra tree classifier, least absolute shrinkage and selection operator (LASSO), and elastic net with different L1 weighting factors [20]. The selected features were then used to train the ML models under two scenarios: using only CT image radiomic biomarkers (radiomics model) and combining clinical, dosimetric, and radiomic features (combined model), to evaluate the contribution of each biomarker in predicting VCD.

**Figure 1:**
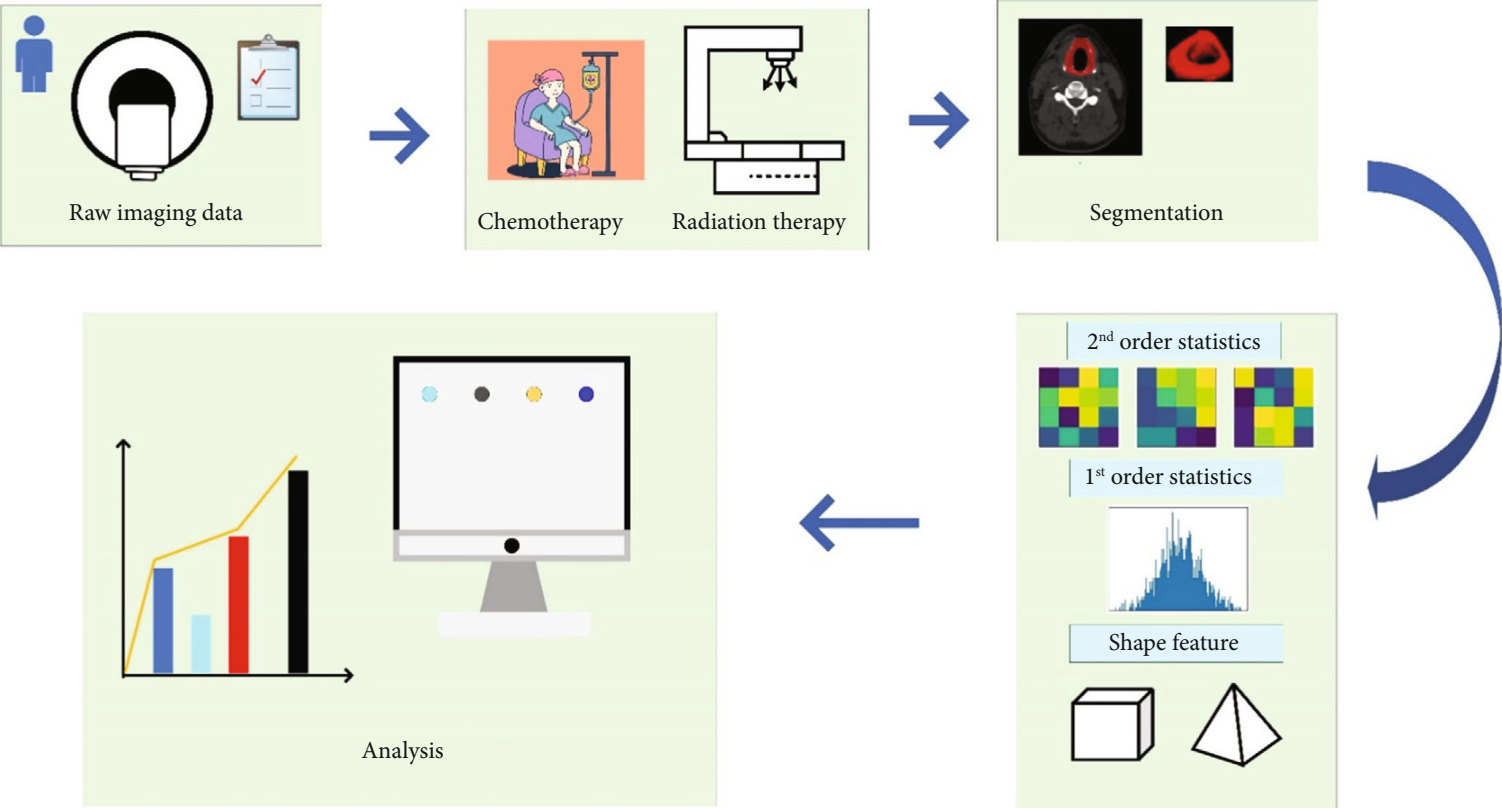
The patient journey appears to involve various steps, including image acquisition, patient‐specific therapy and prognosis, radiomics workflow such as segmentation, feature extraction, feature selection, and analysis utilizing ML algorithms.

The performance of each model was measured using metrics including accuracy, sensitivity, specificity, and the area under the receiver operating characteristic curve (AUC‐ROC). The models are aimed at predicting VCD, which was encoded as a binary outcome (yes = 1 and no = 0). This classification was determined based on acoustic analysis of voice signals and medical examinations. Voice evaluations were independently performed by two experienced speech therapists, each blinded to the other′s assessments. Both experts used the GRBAS scale (grade, roughness, breathiness, asthenia, and strain) to rate the severity of voice impairment. The results were compared, and interrater reliability was assessed using the intraclass correlation coefficient (ICC), which demonstrated excellent agreement (ICC = 0.921) [21]. Radiation‐induced damage was evaluated according to the Common Terminology Criteria for Adverse Events (CTCAE) Version 5.0. VCD was modeled using a range of clinical, dosimetric, and radiomic variables, including chemotherapy, age, sex, smoking status, radiation dose (maximum, minimum, and total), dose–volume histogram (DVH) data, and CT‐derived radiomic features. Nine different classifiers were employed to perform the classification: support vector machine (SVM), logistic regression (LR), random forest (RF), *k*‐nearest neighbor (*k*‐NN), multilayer perceptron, AdaBoost, Naive Bayes, quadratic discriminant analysis, and Gaussian process. To optimize the hyperparameters of the SVM classifier, a grid search method was applied. This involved tuning the kernel type and its free parameters, such as C and *γ* for the radial basis function (RBF) kernel. For the RF classifier, the optimal number of estimators and the number of features to consider at each split were determined using various impurity metrics, with the out‐of‐bag (OOB) error serving as the evaluation criterion [22] as follows:

(1) EOOB=1N∑i=1N1zi∑jxi,yi∉Diℓhjxi,yi, ∨zi=∑jxi,yi∉Di1,

where *N*, *j*, and *z*_*i*_ represent several data points, trees for which the given sample (*x*_*i*_, *y*_*i*_) is an OOB sample, and the number of trees in the RF for which this data point *i* was OOB. For each OOB data point (*x*_*i*_, *i*), the *j*‐th tree *h* *j*(*x*_*i*_) makes a prediction on input *x*_*i*_, which causes a loss measured by the binary cross‐entropy metric *ℓ*. The framework of all procedures is presented in Figure 1.

## 3. Results

Among the 65 patients included in the study, 31 were diagnosed with VCD based on laryngoscopy evaluation and acoustic analysis of voice signals. LR analysis demonstrated the association between demographic and clinical variables and VCD. As shown in Table 2, chemotherapy and laryngeal mean dose had a statistically significant effect on VCD (*p* < 0.05). Based on the dosimetric data, the mean radiation dose delivered to the larynx was 44.8 Gy, with a range of 15–66 Gy. A mean dose exceeding 44 Gy has previously been associated with Grade II or higher laryngeal edema [17].

**Table 2:** Results of logistic regression (LR) analysis to estimate the relationship between demographic, clinical, and dosimetric data with VCD, 6 months after chemoradiation therapy (CRT).

**Variables**	**β**	**OR**	**95% CI (OR)**	**p** **value**
Age	−0.085	0.919	0.190–4.452	0.913
Gender	0.095	1.100	408–2.967	0.851
Smoking	1.187	3.276	0.628–17.079	0.159
Chemotherapy	1.762	5.823	1.620–53.354	0.031 ^∗^
Mean dose	2.231	9.313	1.335–32.354	0.012 ^∗^
Max dose	1.081	2.978	0.388–22.395	0.296

Abbreviations: CI, confidence interval; OR, odds ratio; VCD, vocal cord dysfunction.

^∗^Significant at the *p* < 0.05 level in multivariate analysis.

**Figure 2:**
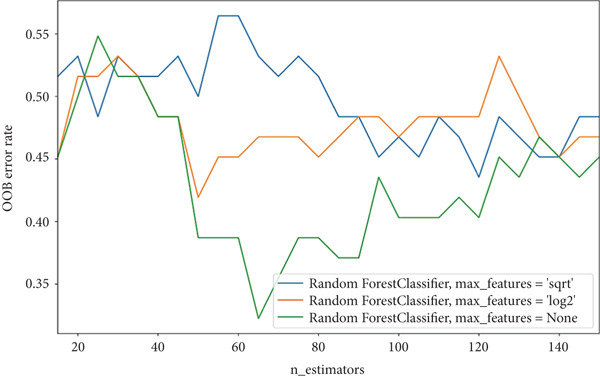
presents the OOB error for the features by the extra tree feature selection method. Three different splitting numbers, including “sqrt,” “log2,” and “all features (None),” were used to plot the classification error.

To predict VCD using radiomic features extracted from CT images, three ML‐based feature selection algorithms and nine different classifiers were applied. The LASSO model selected two radiomic features: one from the GLCM and one from the GLSZM. In contrast, the extra tree classifier selected 40 radiomic features, including eight first‐order statistics, six GLCMs, eight GLSZMs, four GLRLMs, four GLDMs, two NGTDMs, and eight shape features. Lastly, the elastic net with varying L1 weighting factors selected a single texture feature from the GLSZM group. Notably, all three feature selection methods identified a GLSZM texture feature as a predictor of VCD. A grid search method selected the SVM classifier with a sigmoid kernel (SVM‐Sigmoid) as the most effective approach for predicting VCD based on CT radiomic features. Figure 2 presents the OOB error plot for the extra tree feature selection method, illustrating how the number of estimators and features influences RF classification performance. Figures 3a, 3b, and 3c display the AUC curves of the classifiers for features selected by the three algorithms: extra tree classifier, LASSO, and elastic net. Initially, using the features selected by the LASSO algorithm, the AdaBoost and RF classifiers outperformed the other models, achieving AUC values of 0.84 and 0.75, respectively, as shown in Figure 3a. In contrast, the SVM‐Sigmoid classifier had the lowest AUC value of 0.63. When considering the accuracy metric, both SVM‐Sigmoid and 3‐NN classifiers performed equally well, each achieving an accuracy rate of 73.7%. On the other hand, the Gaussian Naive Bayes classifier exhibited the poorest performance, with an accuracy rate of 57.9% (Table 3). For the models trained on features selected by the extra tree classifier, the RF classifier achieved the highest AUC value of 0.69, while the Gaussian process classifier had the lowest, with an AUC of 0.35 (Figure 3b). The accuracy results followed a similar trend, with RF having the highest accuracy of 63.2% and the Gaussian process having the lowest accuracy of 47.2% (see Table 3). Furthermore, for models trained on features selected by the elastic net algorithm, AdaBoost and LR achieved the best performance, each with an AUC of 0.74, whereas SVM had the lowest AUC of 0.43 (Figure 3c). The accuracy metrics reflected a similar pattern: RF and AdaBoost both achieved an accuracy of 63.2%, while SVM‐Sigmoid had the lowest accuracy of 53.2% (Table 3).

Figure 3The ROC curves for different classifiers trained on radiomics features, selected by (a) LASSO, (b) extra tree, and (c) elastic net classifier methods.

**Figure:**
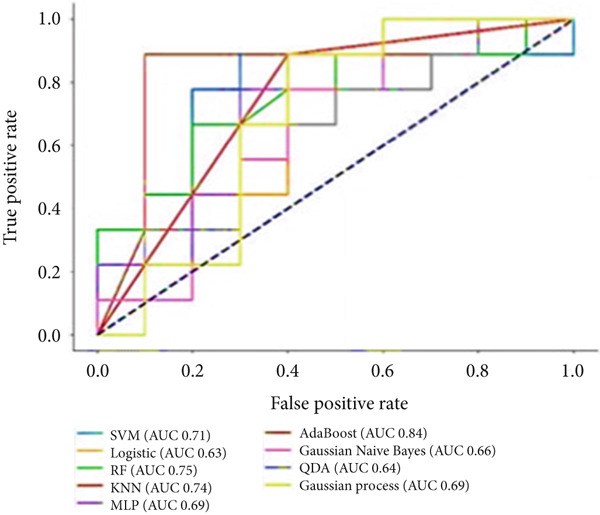
(a)

**Figure:**
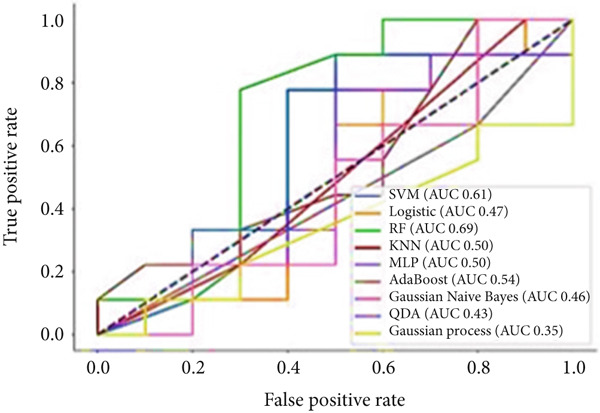
(b)

**Figure:**
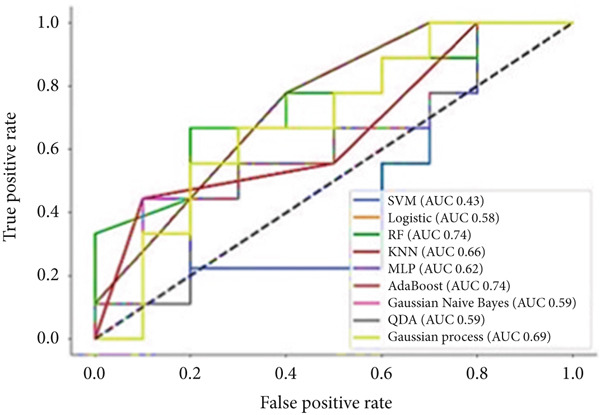
(c)

**Table 3:** The best performance of models based on three algorithms: LASSO, extra tree, and elastic net in radiomics and combined models, respectively.

**Models**	**ML algorithms**	**Classifiers**	**Accuracy**	**Sensitivity**	**Specificity**	**AUC**
Radiomics models	LASSO method	AdaBoost	0.68	0.88	0.80	0.84
	Extra tree	RF	0.63	0.55	0.7	0.69
	Elastic net	AdaBoost	0.63	0.88	0.7	0.74
		RF	0.63	0.88	0.65	0.74

Combined models	LASSO method	SVM	0.95	0.90	0.94	0.99^a^
		RF	0.94	0.88	0.90	0.98^a^
		*k*‐nearest neighbors	0.84	0.77	0.90	0.97^a^
		AdaBoost	0.94	0.91	0.89	0.97^a^
		QDA	0.84	0.77	0.90	0.97^a^
		Gaussian Naive Bayes	0.81	0.85	0.91	0.92
		Multilayer perceptron	0.80	0.77	0.90	0.91
	Extra tree	Multilayer perceptron	0.95	0.88	0.94	0.98^a^
		SVM	0.94	0.90	0.90	0.94
		RF	0.90	0.88	0.91	0.94
		AdaBoost	0.88	0.90	0.92	0.94
		Gaussian process	0.77	0.90	0.88	0.92
	Elastic net	RF	0.98	0.95	0.98	0.99^a^
		SVM	0.92	0.98	0.90	0.98^a^
		*k*‐nearest neighbors	0.93	0.90	0.90	0.97^a^
		Multilayer perceptron	0.99	0.90	0.90	0.97^a^
		AdaBoost	0.93	0.90	0.88	0.93
		Gaussian Naive Bayes	0.89	0.92	0.90	0.92

Abbreviations: AUC, area under the curve; ML, machine learning; RF, random forest; SVM, support vector machine; QDA, quadratic discrimination analysis.

^a^AUC greater than 0.95.

### 3.1. Radiomics, Dosimetric, and Clinical (Combined Model) Results

In this step, radiomic features, dosimetric biomarkers, and clinical factors were combined to predict VCD. The LASSO method selected eight features, including three texture features, one shape feature, and four dosimetric features. The classifiers showed marked improvement in their performance after incorporating both dosimetric and clinical data. For example, the accuracy of the SVM‐Sigmoid classifier increased by approximately 24% compared to the scenario using only radiomic biomarkers (Figure 4a). The extra tree classifier selected 20 features, consisting of four first‐order statistics, 12 texture features, and four dosimetric features. All classifiers showed improved classification results, except for quadratic discriminant analysis, which did not exhibit significant gains. After incorporating dosimetric and clinical data, the accuracy of the RF classifier increased from 63.2% to 93.8%. The LR classifier showed the most substantial improvement, with a 2.2‐fold increase in accuracy (from 36.8% to 81.2%). The AUC values also showed similar enhancements, as illustrated in Figure 4b. The elastic net feature selector chose only dosimetric features, including mean, total, maximum, and minimum dose. This led to improved ML prediction accuracy across most classifiers, with the exception of quadratic discriminant analysis. By incorporating both dosimetric and clinical data, the accuracy of the SVM‐Sigmoid and RF classifiers increased from 52.6% and 47.4%, respectively, to 93.8%. Additionally, the AUC values of all four classifiers exceeded 0.94, as shown in Figure 4c. The performance of each model was evaluated using sensitivity, specificity, accuracy, and AUC metrics, which are summarized in Table 3.

Figure 4The ROC curves for different classifiers trained on radiomic, dosimetric, and clinical features selected by (a) LASSO, (b) extra tree, and (c) elastic net classifiers.

**Figure:**
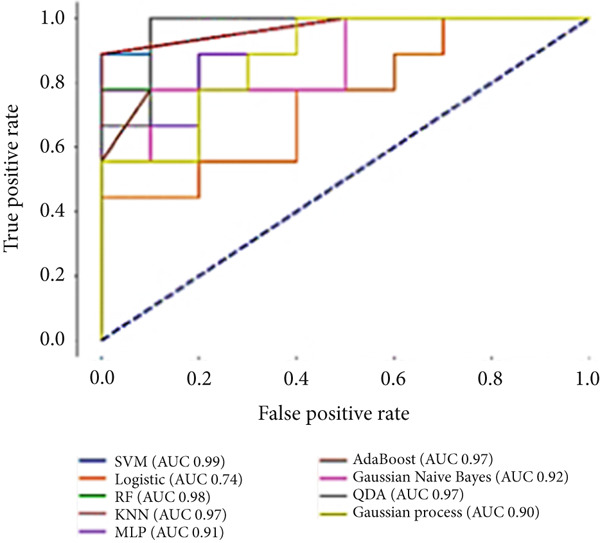
(a)

**Figure:**
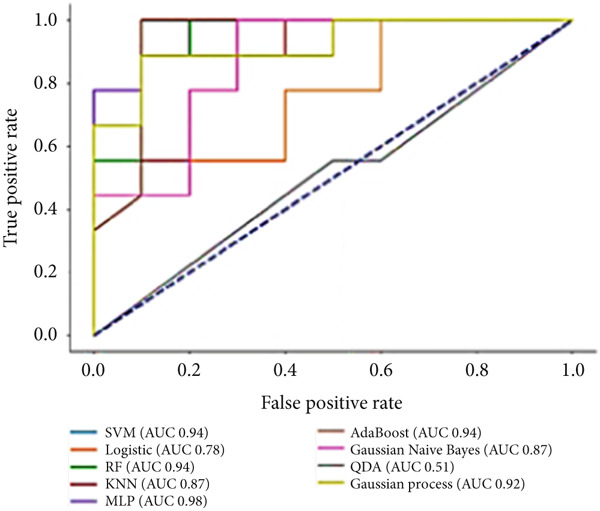
(b)

**Figure:**
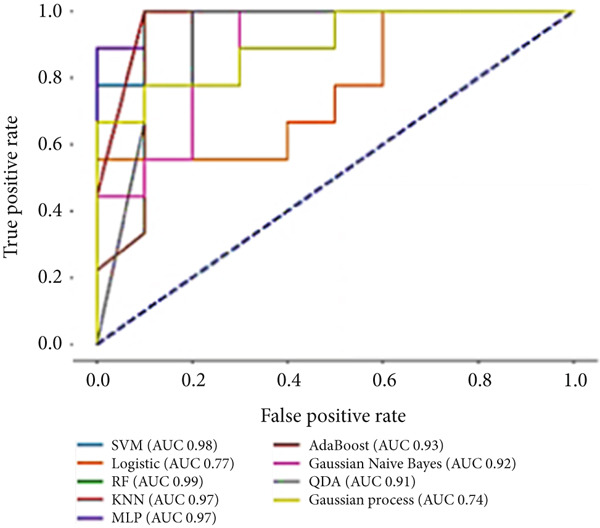
(c)

## 4. Discussion

Voice is a fundamental aspect of social and emotional communication; therefore, vocal problems in patients with HNCs can significantly affect their quality of life and social interactions. Several studies have investigated voice disorders and laryngeal damage following RT [12–15, 21]. The unique contribution of the present study lies in the development of CT‐based radiomic models that integrate clinical and dosimetric data to predict VCD. We hypothesized that combining CT radiomic features with dosimetric and clinical variables would enhance the predictive performance of ML models for VCD. Our results using LR indicated that chemotherapy and the mean laryngeal radiation dose were significant predictors of vocal cord impairment. These findings are consistent with previous studies, which have shown that concurrent chemotherapy exacerbates radiation‐induced toxicity and that a mean radiation dose exceeding 45 Gy increases the risk of vocal cord edema [17, 23, 24]. In this study, ML algorithms were employed to select CT radiomic features for constructing predictive models of VCD. All three ML algorithms consistently identified texture‐based biomarkers, including GLCM and GLSZM features, which indicated reduced symmetry and increased heterogeneity in the imaging data [7, 25]. Our results suggest that GLSZM‐derived features, which quantify the size and distribution of homogeneous intensity zones in CT images, are sensitive to radiotherapy‐induced microstructural changes in the larynx and vocal folds. Radiation to nonlaryngeal head and neck structures can cause edema, inflammation, and necrosis [26], altering tissue spatial heterogeneity, a change effectively captured by GLSZM features. Consistent with prior studies, quantitative texture alterations, such as variations in homogeneity, cluster prominence, and large‐area emphasis, may reflect tissue remodeling processes including fibrosis, necrosis, and microstructural disruption. For example, in stereotactic body radiotherapy for lung cancer, GLCM‐derived features exhibited a clear dose–response relationship and outperformed conventional first‐order measures in detecting radiotherapy‐induced lung injury [27]. Similarly, radiomics approaches have reliably distinguished radiation necrosis from tumor recurrence in white matter and brain tumors [28]. These findings highlight the potential of texture‐based radiomic features to serve as sensitive imaging biomarkers of subclinical tissue changes following radiotherapy. Prior studies have shown that radiation‐induced changes in vocal fold tissue are associated with alterations in the acoustic parameters of voice signals [12, 15, 29]. Consequently, alterations in tissue properties captured by CT radiomic features may reflect underlying vocal dysfunction. A comparable study by van Dijk et al. reported a correlation between the “Short Run Emphasis” (SRE) feature from the GLRLM category and late parotid gland toxicity [30]. Our radiomics models demonstrated satisfactory predictive accuracy, with AUC values ranging from 0.69 to 0.84 across all ML algorithms. Previous studies suggest that patient‐specific factors account for approximately 80% of normal tissue radiosensitivity [25]. Moreover, our findings support the potential of pretreatment CT radiomic biomarkers as valuable predictors of VCD in patients with nonlaryngeal HNC. Consistent with previous studies, our results further emphasize that integrating clinical, dosimetric, and demographic data produces the most effective models for predicting radiotherapy outcomes [6, 31, 32]. In the next phase, we developed predictive models that incorporated CT radiomics, dosimetric, and clinical variables, referred to as the combined model. Using the LASSO algorithm, two key features were identified, yielding AUCs ranging from 0.74 to 0.99 across different classifiers. The extra tree algorithm achieved AUCs between 0.51 and 0.97, while the elastic net algorithm produced four models with AUCs exceeding 0.95. Across all algorithms, the combined models consistently outperformed the radiomics‐only models in terms of AUC, sensitivity, and specificity. These findings highlight the critical importance of integrating imaging, dosimetric, and clinical data to predict VCD and radiation‐induced laryngeal injury (Table 3). A key contribution of this study is the application of multiple ML algorithms for feature selection, combined with the development of predictive models using a variety of classification techniques. The robust performance of these models reinforces the validity and reliability of our results. Overall, this study identified significant predictive models for VCD in patients with nonlaryngeal HNC. This is particularly important given that voice analysis and clinical evaluation of vocal cord function are specialized and often invasive procedures [13, 15, 29]. The use of ML‐based prediction models offers a valuable, noninvasive alternative. Our approach represents a cost‐effective method for complication prediction based on pretreatment CT imaging. Similar studies have applied ML models to predict radiation‐induced toxicities in normal tissues or organs at risk (OAR). For example, Mostafaei et al. predicted gastrointestinal and urinary toxicities in prostate cancer patients using pretreatment CT radiomic features, finding that combining radiomics with clinical and dosimetric variables improved predictive power [7]. Likewise, Abdollahi et al. demonstrated that MRI radiomic features were correlated with rectal wall changes following intensity‐modulated radiotherapy (IMRT) in prostate cancer patients, enabling prediction of rectal toxicity [33]. Despite these promising results, our study has limitations. While using sustained /a/ phonation aligns with established acoustic voice assessment protocols, it provides only a partial view of vocal function. Given evidence suggesting that different vowels (e.g., /i/ and /e/) may exhibit distinct acoustic behaviors and diagnostic value [34], future studies should include additional sustained vowels or connected speech tasks to obtain a more comprehensive evaluation of voice quality. We analyzed only CT radiomic features; incorporating imaging biomarkers from additional modalities, such as multiparametric MRI, may enhance model performance and improve our understanding of CRT‐induced vocal dysfunction. Additionally, our ML models were trained on a relatively small clinical dataset, underscoring the need for larger datasets to confirm the generalizability and robustness of our findings. Although long‐term follow‐up could reveal additional changes in vocal function, patient attrition over extended periods was considerable; out of approximately 70 patients, only 30 remained at 18 months posttreatment [29]. To maintain an adequate sample size and ensure valid statistical analysis, we focused on earlier assessment time points (baseline, posttreatment, and 6 months). Nonetheless, we employed rigorous analytical approaches to ensure the validity of our results.

## 5. Conclusion

This study demonstrates that pretreatment CT radiomic features serve as novel biomarkers for predicting radiation‐induced VCD. Furthermore, integrating radiomic, clinical, and dosimetric variables significantly improves predictive models for radiotherapy outcomes such as VCD.

## Conflicts of Interest

The authors declare no conflicts of interest.

## Funding

This study was funded by the Iran University of Medical Sciences, 10.13039/100012021, 1401‐2‐4‐23583.

## Data Availability

The data that support the findings of this study are available on request from the corresponding author. The data are not publicly available due to privacy or ethical restrictions.
